# Comparing surface electroenterography measurements between patients suffering from Hirschsprung’s disease and controls: a feasibility study

**DOI:** 10.1038/s41598-024-54189-4

**Published:** 2024-02-13

**Authors:** Nick Rolleman, Willemijn Klein, Iris Nagtegaal, Michel van Putten, Ivo de Blaauw, Sanne Botden

**Affiliations:** 1 Department of Pediatric Surgery, Radboud University Medical Centre-Amalia Children’s Hospital, Geert Grooteplein Zuid 10, Postal box 9101, 6500 HB Nijmegen, The Netherlands; 2 Department of Medical Imaging, Radboud University Medical Centre, Nijmegen, The Netherlands; 3 Department of Pathology, Radboud University Medical Centre, Nijmegen, The Netherlands; 4 Department of Clinical Neurophysiology, University of Twente, Enschede, The Netherlands

**Keywords:** Functional gastrointestinal disorders, Paediatrics

## Abstract

Current diagnostics in Hirschsprung’s disease are often challenging and invasive. This study aims to investigate whether surface electroenterography can non-invasively discern healthy subjects from subjects suffering from Hirschsprung’s disease. Nine healthy subjects (seven children, two adults) and eleven subjects suffering from surgically untreated Hirschsprung’s disease (nine children, two adults) underwent an electroenterography procedure. This procedure consisted of ultrasound-guided placement of surface electrodes on the abdomen covering all parts of the colon, fasting and two 20-min electroenterography measurements separated by a meal. The dominant frequency, magnitude and relative increase (pre- to postprandial) of colonic activity were compared between both groups. The results showed that in the pediatric group, no significant differences in dominant frequency, colonic activity and relative power increase were observed between controls and patients. The adult patients showed decreased colonic motility and relative power increase in the electrodes closest to the distal colon, both when compared to the same electrodes in controls and to the more proximal electrodes of themselves. To conclude, electroenterography measurements in young children is challenging, but the results in adults demonstrate that these measurements can possibly distinguish between controls and Hirschsprung’s patients. Therefore, optimization of electroenterography measurements in young children is necessary.

## Introduction

With an incidence of 1:5000 live births, Hirschsprung’s disease (HSCR) is the most prevalent congenital colonic motility disorder^1^. HSCR is characterized by the absence of myenteric and submucosal nerve plexuses in the distal colon, leading to functional obstruction^2^. If left untreated, patients are often unable to pass stool at infants age and are at risk of developing severe bowel obstruction and Hirschsprung associated enterocolitis, a potentially fatal inflammatory bowel disorder^3^. Therefore, an adequate and timely diagnosis is paramount for these patients.

The gold standard in diagnosing HSCR is a rectal (suction) biopsy, which can be performed with the patient awake or under general anesthesia^4,5^. However, a recent study has shown that up to one in four biopsies are inconclusive and a re-biopsy is necessary^6^. Another study revealed that only 15% of the patients who underwent a rectal biopsy, as indicated by the U.K. National Institute for Health and Clinical Excellence, suffered from HSCR^7^. This means that approximately 85% of the patients underwent this invasive procedure without receiving a diagnosis. The invasiveness and limited efficacy of rectal biopsies advocate for a non-invasive and more adequate method for the diagnosis of HSCR.

Recently, surface electroenterography (sEEnG), in which surface electrodes are placed on the abdomen to measure the slow wave muscle activity of the colon, has been investigated as a non-invasive alternative to current diagnostics for colonic motility disorders^8^. This slow wave muscle activity of the colon has a dominant frequency between 2 and 6 cycles per minute (cpm) and postprandially increase in number, as shown using high-density manometry^9^. A study in adults without gastrointestinal complaints has demonstrated that sEEnG is able to record the gastrocolic reflex as a measure of colonic activity^8^. However, sEEnG measurements have neither been described in children nor in HSCR patients. The aims of this explorative study were to evaluate the feasibility of sEEnG measurements in healthy children and to investigate the differences between sEEnG recordings in adults and children suffering from Hirschsprung’s disease and controls. Additionally, the patients’ experience of the sEEnG procedure was compared with their experience of the rectal biopsy.

## Methods

### Study design and setting

In this observational, cross-sectional study, pre- and postprandial sEEnG measurements were performed in adults and children, both healthy and suffering from HSCR, to measure the gastrocolic reflex. The study was conducted at the Radboudumc-Amalia Children’s hospital, Nijmegen, the Netherlands. Participants were included from February 2021 to March 2022.

### Participants

Both patients and controls in this study were < 12 or > 18 years old, with the aim to match the age of the controls with the patient group. To avoid biased results due to near adult body posture, teenagers were excluded. Patients were included if they suffered from HSCR as confirmed by a rectal biopsy, for which surgical correction was not yet performed. Exclusion criteria for both groups were a weight-for-length Z-score > 2.5 standard deviations (SD) for children or a BMI > 27 kg/m^2^ for adults, diabetes, any food intolerances, presence of an intestinal stoma, the dependency on continuous tube feeding, previous intestinal surgery and inflammatory bowel diseases. Additional exclusion criteria for the control group were the presence of any gastrointestinal disease or complaint and the use of laxatives in the past two years. Controls were recruited from the Dutch patient organization for Hirschsprung’s disease and from patients visiting the Radboudumc-Amalia Children’s hospital with complaints unrelated to the gastrointestinal tract. For the patient group, all potential eligible patients who visited the Radboudumc-Amalia Children’s hospital during the inclusion period were approached for participation.

### Protocol

**Figure 1:**
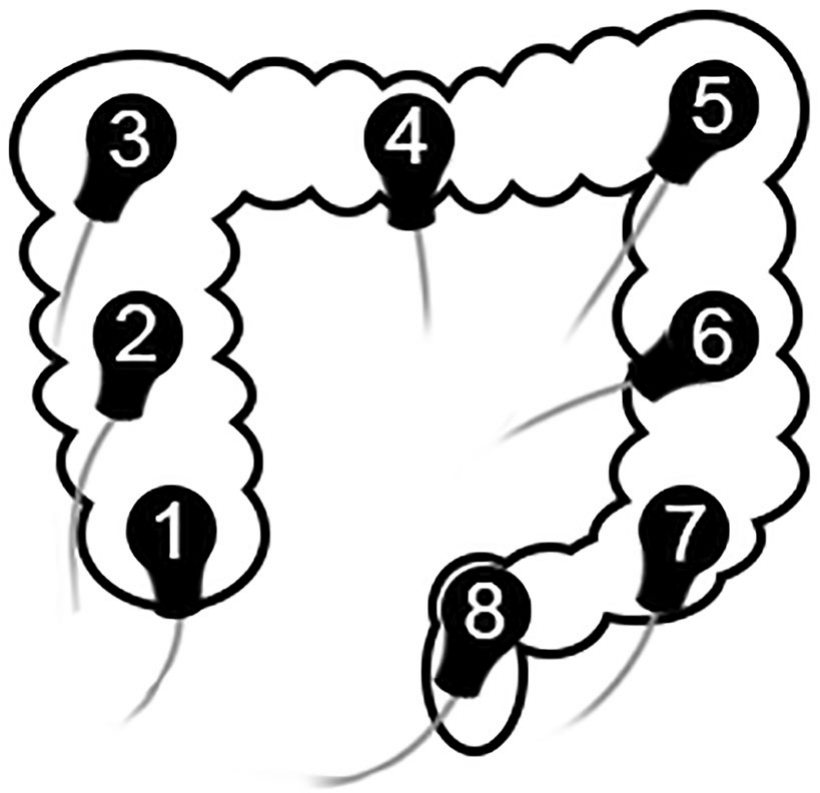
SEEnG electrode positions, spread evenly to cover the entire colon.

Before the sEEnG procedure, participants fasted for at least four hours (three hours for participants under one year old). Next, with the participant in supine position, an experienced radiologist used ultrasound to position eight surface electrodes on the abdomen, see Fig. 1. Subsequently, a 20-min baseline sEEnG recording was performed, after which the participants consumed a meal consisting of at least 1/6th of their daily recommended calorie intake based on the advice of The Netherlands Nutrition Centre, thereby evoking the gastrocolic reflex^10^. Young children drank their usual amount of breastmilk or formula. After the meal, another 20-min sEEnG recording was performed to measure the colonic activity induced by this reflex. The measurements were obtained using silver-silver chloride electrodes (30 × 24 mm, Covidien, Massachusetts, USA), a DC-coupled amplifier, suitable for measuring low frequency signals, (Porti7, TMSi, Oldenzaal, the Netherlands) and the Polybench software (version 1.34.0, TMSi, Oldenzaal, the Netherlands). The signals were sampled at 2048 Hz and were measured using a unipolar configuration with average reference amplification. Additionally, participant characteristics (age, sex, weight, length and weight-for-length Z-score or BMI) and defecation habits (defecation frequency, average Bristol stool scale^11^ and time since last defecation) were obtained. In HSCR patients who underwent surgical treatment after participation, the aganglionic length of the colon, as determined by the pathologist, was also obtained.

### Signal analysis

The preprocessing steps of the sEEnG recordings included downsampling to 20.48 Hz and a 4th order Butterworth bandpass filter between 1.5 and 10 cpm to ensure all activity between 2 and 6 cpm is completely incorporated. Additional artifact removal was performed using the algorithm proposed by Gharibans et al.^12^. Next, the signals were translated to the frequency domain using Welch’s method using 240-s Hann windowing with 50% overlap. From the resulting spectrograms, three features were determined. First, the frequency with the highest power density was denoted as the dominant frequency. Then, as a measure of colonic activity, the mean power density (MPD), expressed in µV^2^/cpm, was defined by the average power density in a 2-cpm bandwidth around the dominant frequency. Lastly, the power percent difference (PPD), the relative increase in the MPD between the pre- and postprandial recordings, was calculated. These features were averaged over all electrodes as well as calculated for each individual electrode position.

To assess the feasibility of the sEEnG measurements in children, the differences between the pre- and postprandial sEEnG features were evaluated in the pediatric control group. The ability of sEEnG to distinguish between HSCR patients and controls was based on differences between both groups as well as on interelectrode differences within the patient group.

### Patient experience

To investigate the experiences of the sEEnG procedure with respect to the rectal biopsy, the parents of pediatric HSCR patients completed a questionnaire about both of these procedures. The questionnaire was created in cooperation with a pediatric surgeon, a pediatric psychologist and representatives of the Dutch patient organization for Hirschsprung’s disease. It contains questions regarding prior expectations, pain, duration and burden of the procedure, see Appendix A. The questions could be scored from zero (most negative) to ten (most positive).

### Statistical analysis

All values are presented as mean ± standard deviation or median (interquartile 1–interquartile 3), as appropriate. Differences between pre- and postprandial sEEnG features in the pediatric control group were tested using Wilcoxon signed-rank tests. Differences in participant characteristics and average sEEnG features between pediatric controls and patients were tested using Pearson’s Chi-square test or Mann–Whitney U tests. Differences in electrode-specific sEEnG features between pediatric controls and patients were tested using the median test. The sEEnG results of the adult groups were analyzed in a descriptive manner, using the sEEnG features as described above. The pre- and postprandial dominant frequency and MPD, averaged over all electrode locations, were compared between healthy adults and patients, as well as the pre- and postprandial MPD on the individual electrode level. The results of the questionnaires completed by parents of patients were compared using Wilcoxon matched-pairs signed-rank tests. For all tests, p-values < 0.05 were considered statistically significant.

### Ethical approval

Approval from the local medical ethical committee (CMO region Arnhem-Nijmegen) was obtained (NL75302.091.20) and the study was performed in accordance with the ethical standards laid down in the 1964 Declaration of Helsinki. All subjects (or their parents) signed informed consent prior to participation.

## Results

In total, sixteen children and four adults were included in the study. Due to the low number of adults, their results will be analyzed descriptively. The pediatric group consisted of seven controls and nine patients, of which four were girls. On average, they were 1.6 years (range: 0.2–11) old and had a weight-for-length Z-score of 0.3 SD (range: − 1.8 to 2.2). Table 1 shows the participant characteristics per group. The controls tended to be older than the patients, but this difference was not significant (*p* = 0.055). Furthermore, in the control group, the BSS was lower (*p* = 0.042) and the meal content higher (*p* = 0.042). The aganglionic segment in all patients was confined to the rectosigmoid colon, with a medianaganglionic length of 5.2 cm. The other characteristics did not differ between the groups. In one participant in the control group, electrode eight was not attached properly during the postprandial measurement. This was also the case during the preprandial recording in one patient. In two other participants, one of each group, the 20-min preprandial recording time was not completed due to logistical reasons. The other subjects fully completed the sEEnG procedure.

**Table 1:** Participant characteristics of both pediatric groups.

	Controls (n = 7)	Patients (n = 9)	*p*-value
Girls (n)	3	1	0.146
Age (years)	1.5 (0.5–5.0)	0.3 (0.3–0.4)	0.055
Weight-for-length Z-score (SD)	0.3 (-0.5–1.3)	0.5 (-0.2–0.7)	0.918
Defecation frequency (per week)	10 (7–17)	14 (14–15.5)	0.210
Bristol stool scale^a^	5 (4–6)	6 (6–7)	0.042
Duration of fasting (h)	4.0 (3.0–4.5)	3.5 (3–4.5)	0.837
Meal content (kcal)	222 (112–492)	101 (79–142)	0.042
Meal duration (min)	10.3 (6.0–13.6)	17.2 (4.1–24.8)	0.606
Aganglionic length (cm)	–	5.2 (3.5–6.5)	–

^a^n = 4 for the patient group, as the other patients completely relied on rectal irrigation.

### Pediatric controls

Among the pediatric controls, the preprandial dominant frequency was 3.3 cpm (3.0–3.9) and did not differ from the postprandial dominant frequency 3.2 cpm (3.0–3.4), *p* = 0.310. The MPD, however, tended to be higher in the postprandial recordings, with pre- and postprandial values of 974 µV^2^/cpm (393–4646) and 5474 µV^2^/cpm (1153–5958) respectively, but statistical significance was not reached (*p* = 0.091). The PPD, representing the relative increase in MPD, was 269% (8–384).

### Pediatric controls vs. patients

**Figure 2:**
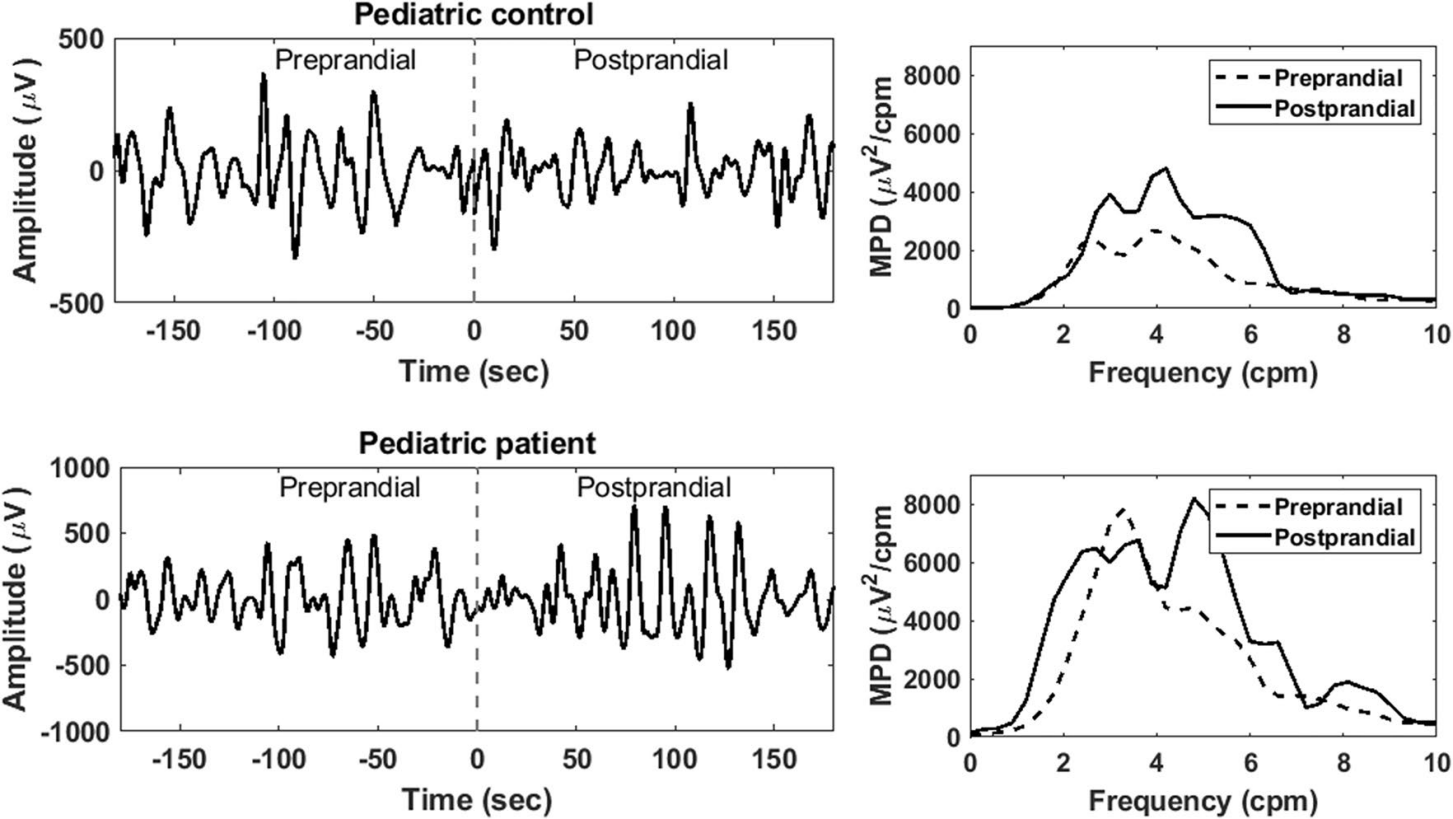
Representative sEEnG tracings and corresponding frequency plots of pediatric controls (top) and pediatric patients (bottom) of electrode 8.

Representative sEEnG signals and corresponding frequency plots of pediatric controls and patients are shown in Fig. 2. Averaged over all electrodes, the dominant frequency in the pediatric patients did not differ from the controls, with a mean difference of 0.3 ± 0.2 cpm for both the pre- and postprandial values. The MPD did also not differ between the groups, with differences between controls and patients of 1944 ± 2971 µV^2^/cpm, *p* = 0.536, for the preprandial recordings and 428 ± 2116 µV^2^/cpm, *p* = 0.918, for the postprandial recordings. Consequently, the PPD was also comparable between the groups, with a mean difference of -101 ± 163%, *p* = 0.606.

**Figure 3:**
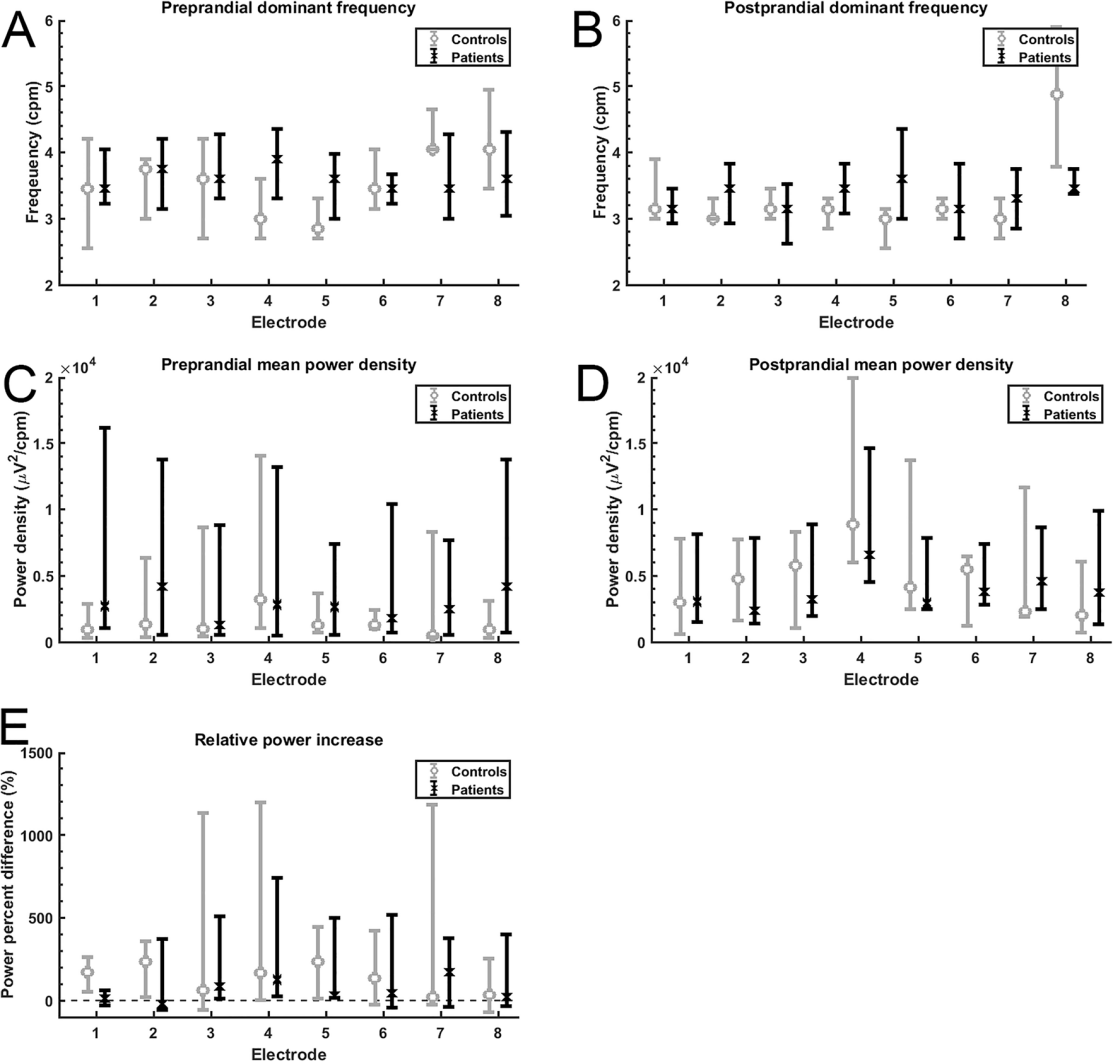
(**A**,**B**) Pre- and postprandial dominant frequencies per electrode for pediatric controls (grey, N = 9) and patients (N = 7).. Note that the postprandial dominant frequency at electrode eight is significantly higher in controls when compared to patients. (**C**,**D**) Pre- and postprandial MPD’s per electrode per group. The medians for the pre- and postprandial values are comparable, but the interquartile range, especially in electrode one and eight, is wider in the patient group. E: Relative power increase per electrode per group. The PPD at electrode 1 is significantly lower in the patient group when compared to controls.

Because HSCR is, in most patients, confined to the distal part of the colon, the sEEnG features were also studied per electrode position. Figure 3 shows each feature per electrode for the control and the patient group. Concerning the dominant frequency, no differences between the groups were observed and nearly all pre- and postprandial values for both groups are well within the 2–4 cpm bandwidth. However, postprandial electrode eight in the control group differed significantly from the patients (*p* = 0.011). Visual inspection of the corresponding power spectra showed that for most of these cases, no clear power peak at any frequency was present. Figure 3C demonstrates that the interquartile range of the preprandial MPD in patients was wider than the interquartile range in controls for more than half of the electrode positions, although this was most pronounced at the caudal electrodes, electrodes one and eight. The medians were comparable for both groups, which was also true for the postprandial MPD. The PPD’s of both groups were also comparable for electrodes, except electrode one, which shows a lower PPD in the patient group when compared to the controls (*p* = 0.041). In the patient group, none of the features derived from the distal electrodes, targeted at the aganglionic segment, differed from the more proximal electrodes.

### Adults controls vs. adult patients

In the adult group, two controls and two patients were included and each group consisted of one man and one woman. They were 44 years (range: 39–47) old and had a BMI of 24 kg/m^2^ (range: 22.6–26.5). They fasted 4.3 h (range: 4–5) before the sEEnG procedure, consumed 542 kcal (range: 290–899) in between the sEEnG measurements and the average meal duration was 17.4 min (range: 6.5–27). Both patients relied fully on rectal irrigation and the exact length of aganglionosis was not known. All adult participants fully completed the sEEnG procedure.

**Figure 4:**
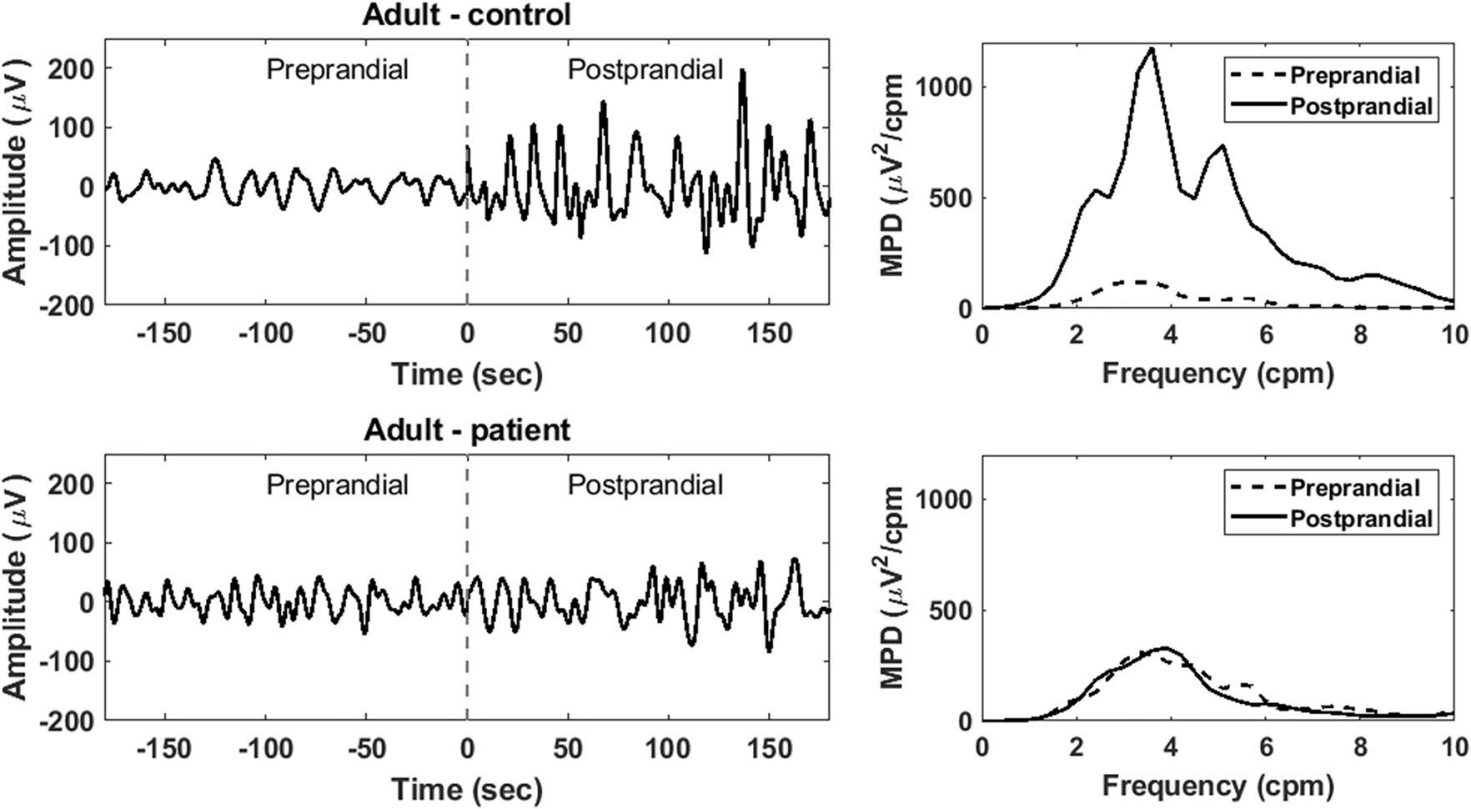
Representative sEEnG signal tracings and corresponding frequency plots of adult controls (top) and adult patients (bottom) of electrode 8.

**Figure 5:**
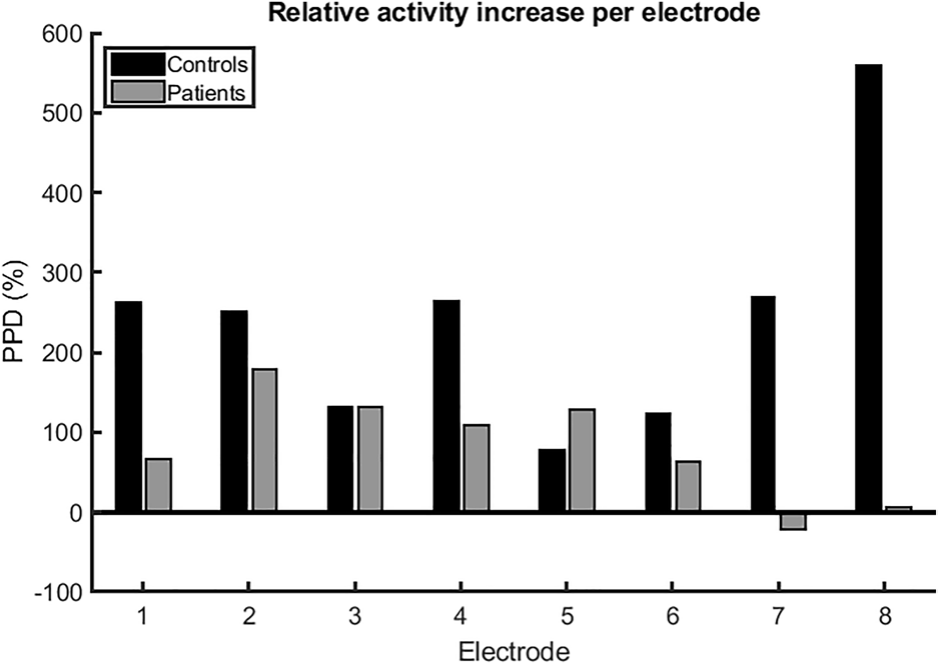
PPD per electrode of controls (black, N = 7) and patients (grey, N = 7). The PPD of the distal electrodes (seven and eight) is clearly lower compared to the same electrodes in the control group as well as compared to the more proximal electrodes in the patient group.

Representative sEEnG tracings and corresponding frequency plots of both controls and patients are shown in Fig. 4. Averaged over all adults, the dominant frequency was 3.8 cpm (range: 3.1–4.8) for the preprandial recordings and 3.5 cpm (range: 3.2–4.4) for the postprandial recordings. No evident differences between controls and patients was observed. The average preprandial MPD was 350 µV^2^/cpm in controls and 200 µV^2^/cpm in patients and respectively increased to 1108 µV^2^/cpm and 373 µV^2^/cpm postprandially. In Fig. 5, the PPD per electrode per group is presented. It is clear that the PPD in the distal electrodes (seven and eight) of the patient group is lower compared to these electrodes in the control group. Additionally, the PPD in the distal electrodes is also evidently lower than the more proximal electrodes within the patient group.

### Questionnaires

Parents of all patients completed the questionnaires about both the sEEnG procedure and the procedure with which their child was diagnosed. Parents of one patient were unable to fully complete the questionnaire about the rectal biopsy, because they were not present during the procedure. Averaged over all questions, the sEEnG procedure was scored higher than the gold standard procedure (8.9 (8.1–9.9) vs. 5.3 (3.4–7.7), *p* = 0.017). Analysis per question demonstrated that all questions, except for question two regarding the understanding of the procedure, scored significantly higher for the sEEnG procedure than for the rectal biopsy. See Appendix B for the average scores per question.

## Discussion

In this study, pre- and postprandial sEEnG measurements were performed in healthy participants as well as in patients suffering from HSCR. The results showed no statistically significant differences between pre- and postprandial sEEnG recordings in healthy children. Furthermore, there were no clear differences in any of the sEEnG features between pediatric controls and patients. On the contrary, adults with HSCR clearly exhibited lower postprandial activity in the distal colon when compared to healthy adults, corresponding to their aganglionic colonic segment. Besides, parents of pediatric patients experienced the sEEnG procedure to be less burdensome than a rectal biopsy. The current sEEnG protocol seems to be able to distinguish between adult controls and HSCR patients, but it is inadequate for young children. Nevertheless, the questionnaire outcomes underline the need for a less burdensome method to diagnose HSCR.

This study is the first to explore sEEnG measurements in children and to compare sEEnG measurements between HSCR patients and controls. In the pediatric controls, postprandial colonic activity was not significantly higher than the preprandial colonic activity. This contradicts previous sEEnG studies in healthy adults, which have demonstrated a clear postprandial increase in colonic activity when compared to preprandial measurements^8,13^. Furthermore, in the pediatric group, no clear differences between the controls and patients were observed. Earlier research, using intraluminal EEnG measurements, has shown that children suffering from HSCR exhibited no slow wave colonic activity as opposed to healthy controls, in whom slow wave colonic activity was present^14^. Additionally, Dorfman et al. documented an increase in colonic activity upon meal intake in children using colonic manometry recordings^15^. Our contradicting results may be explained by the fact that newborns and young children were not capable to lie completely still during both 20-minte sEEnG recordings. This induced significant movement noise in the recordings, hampering adequate signal analysis. Additionally, 20-min pre- and postprandial recording durations may not be sufficiently long to adequately capture the fasting state and the postprandial response. Rao et al. used 24-h ambulatory manometry recordings to demonstrate that several colonic movement patterns can occur seemingly random, meaning that a 20-min recording may not really reflect a typical fasting state^16^. Therefore, based on our data, the ability of surface electrodes to adequately record colonic activity in the pediatric population cannot be confirmed nor denied. The adult patients, however, showed a distinctly lower postprandial activity increase in the distal colon when compared either to the controls or their own proximal colon. Previous studies in HSCR patients, using intraluminal electrodes, have provided results that are in line with our findings in the adult group^14,17^. They demonstrated a flattening or absence of the colonic slow waves among HSCR patients, corresponding to the aganglionic segment of the colon. Our study provided new insights in the use of sEEnG in young children and to the possibilities and challenges in distinguishing between HSCR patients and controls.

The study’s main strength is the focus on HSCR instead of other colonic motility disorders to assess the use of sEEnG as a diagnostic tool. Because HSCR is an organic disorder, the affected part of the colon can be exactly determined, whereas this is much harder in non-organic colonic motility disorders. Besides, positioning the electrodes using ultrasound-guidance allowed for an analysis of activity per colonic segment, which is paramount in the diagnosis of HSCR. Another strength of our study is that adult patients with HSCR were included, even though there were just two. Adults, as opposed to young children, can be well instructed to lie still, which allowed to assess the technical and physiological feasibility of using sEEnG in the diagnosis of HSCR. However, caution is advised for the direct comparison of pediatric and adult subjects, as several gut functions, including colonic motility, differ between young children and adults^18^. A limiting factor of our study was that the pediatric patients largely consisted of newborns, whereas the controls’ age was more heterogenous. Subsequently, calorie intake was significantly higher in the pediatric control group compared to the patients, possibly influencing the comparison between pediatric controls and patients. Secondly, during the ultrasound-guided positioning of the electrodes, it was observed that the distance between the colon and abdominal wall varied within individuals and that other organs, such as the liver or small intestine, sometimes overlapped the colon. However, by applying a low-pass filter at 10 cpm, interference of the small intestine, which has dominant frequencies between 8 and 12 cpm, is minimized^19^. The varying distances between the colon and the abdominal wall and overlap from non-muscular organs presumably only affected the amplitude of the measured signal and is not relevant in intra-subject comparisons. Nevertheless, this may have impacted the inter-subject comparisons. Another potential limitation of our study is that the number of pediatric participants, especially in the control group, was probably too low to detect significant differences between the pre- and postprandial colonic motility. In the adult population, the subjects consumed a meal of their own choice in between the pre- and postprandial sEEnG recordings. This led to a varying calorie intake between adults, which may impact the reproducibility of the study.

Despite the limitations, valuable lessons can be learned from this study. First, adequate 20-min pre- and postprandial sEEnG measurements in children are challenging due to their inability to lie still during the measurements. These movements have presumably contaminated the signals to such extent, that the colonic signals are overshadowed by the resulting noise. This might explain that the sEEnG features did not differ between pediatric controls and patients and that there were also no interelectrode differences in the pediatric patient group. In addition, noise due to subject movements may also be accountable for the absence of clear postprandial power peaks in electrode eight in the pediatric controls. This electrode is the closest to the legs, which presumably are the most culpable for the movement noise. Following the same reasoning, the wide interquartile range of the pediatric patients’ preprandial MPD in electrode one and eight, both close to the legs, and the patients’ low PPD of electrode one can presumably be explained. It was also observed that some pediatric subjects moved much more during the preprandial recording compared to the postprandial recording, assumably due to hunger. This may explain the negative values of the PPD observed in some patients, as shown in Fig. 2. However, the contribution of physiological differences in colonic motility between both groups to these findings cannot be fully excluded based on our data. Secondly, our data in the adult population suggests the used sEEnG protocol might be able to locally assess colonic motility, as clear differences between controls and patients were present in the distal electrodes. A third lesson this study taught, is that the parents of patients preferred the sEEnG method over rectal biopsies. The questionnaires revealed that parents preferred the sEEnG method over a rectal biopsy on almost all facets of the procedure.

Future research should aim to optimize the sEEnG protocol to improve its applicability in children. Wireless electrode patches, as described by Axelrod et al., may aid in this process^20^. Such patches allow for continuous ambulatory monitoring up to several days, meaning adequate sEEnG signals can be selected during quiet periods such as during sleep, thereby eliminating signal parts contaminated by noise. Aside from HSCR, sEEnG may also be of value in the diagnosis of other colonic motility disorders and should therefore be focused on in further studies.

To conclude, this study showed that sEEnG measurements in children is challenging and did not show differences between pre- and postprandial measurements or patients and controls. In adults, on the other contrary, sEEnG seems a promising technique to assess colonic motility, albeit that just two subjects per group were included. Therefore, further study to optimize the measurement setup is the first necessary step in the development of sEEnG as a non-invasive technique to assess colonic motility.

### Supplementary Information

Supplementary Information 1.

Supplementary Information 2.

## Data Availability

Data are available from the author upon reasonable request by sending an email to nickrolleman@outlook.com.
