# Supplementary material for: Comparing surface electroenterography measurements between patients suffering from Hirschsprung’s disease and controls: a feasibility study
Source: Sci Rep. 2024 Feb 13;14:3585. doi: 10.1038/s41598-024-54189-4 (PMC10864271; doi:10.1038/s41598-024-54189-4)
Supplement: Supplementary file 1 — Supplementary Information 1. [file 41598_2024_54189_MOESM1_ESM.docx]

**Appendix A: Questionnaire diagnostic procedure**

For all questions, a score between 0 to 10 can be given. The meaning of 0 and 10 is described at each question.

**1a. To what extent were you anxious before the procedure for yourself?**

*(0 = very much, 10 = not at all)*

**1b. To what extent were you anxious before the procedure for your child?***

*(0 = very much, 10 = not at all)*

**2. To what extent was the procedure in advance clear to you?**

*(0 = completely unclear; 10 = completely clear)*

**3. To what extent did you find the duration of the procedure burdensome for your child?**

*(0 = very burdensome; 10 = not burdensome at all)*

**4. Do you think your child experienced pain during the procedure?**

*(0 = very much; 10 = not at all)*

**5. To what extent do you think your child experienced the procedure as burdensome?**

*(0 = very burdensome; 10 = not burdensome at all)*

**6. How would you rate the procedure in general?**

*(0 = very burdensome; 10 = not burdensome at all)*

Note: the original questionnaire was in Dutch, but was translated for this appendix.
