# Supplementary material for: Comparing surface electroenterography measurements between patients suffering from Hirschsprung’s disease and controls: a feasibility study
Source: Sci Rep. 2024 Feb 13;14:3585. doi: 10.1038/s41598-024-54189-4 (PMC10864271; doi:10.1038/s41598-024-54189-4)
Supplement: Supplementary file 2 — Supplementary Information 2. [file 41598_2024_54189_MOESM2_ESM.docx]

**Appendix B: Procedure questionnairs: scores per question**


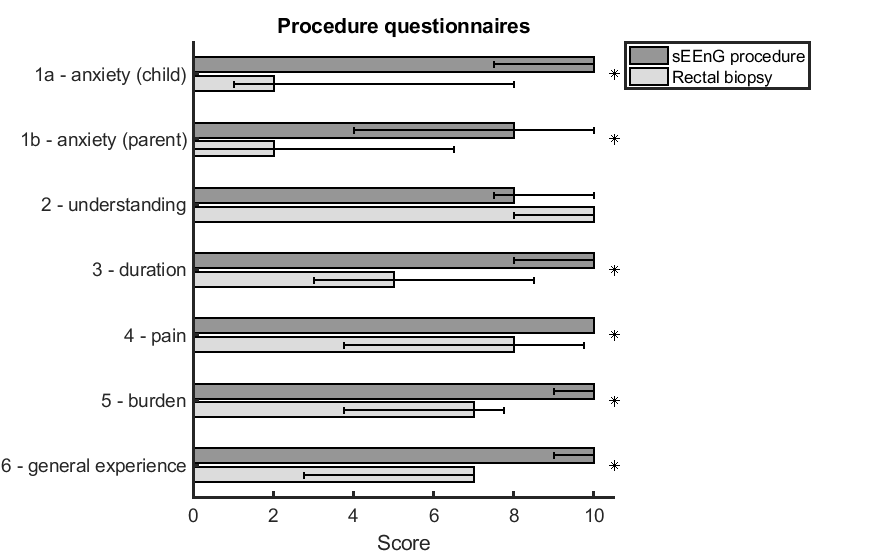


**p* <0.05

**Fig. 4** Questionnaire scores for sEEnG procedure (dark grey) and rectal biopsy (light grey). Scores range from 0 (negative experience) to 10 (positive experience). For all questions, except question 2, the sEEnG procedure was scored significantly higher than the rectal biopsy
